# The Gastric Function

**Published:** 1920-09-25

**Authors:** 


### THE GASTRIC FUNCTION.

A short time ago we read with great interest an article coming from the pen of Dr. J. A. Ryle, assistant physician to Guy's Hospital, which appeared recently in the *Lancet*. His subject, the investigation of the gastric function by means of the fractional test meal, is one of singular importance in everyday medicine.

Not that the method provides the practitioner with a short cut to immediate diagnosis, for the lessons it teaches appear if anything rather to complicate matters from this point of view. The outstanding point is, however, that in the fractional test-meal we apparently have a clinical method which is *really* going to teach the medical profession some definite and precise details concerning the normal and abnormal mechanism of the gastric and duodenal tracts: In the past—and the text-books are largely to blame—far too much reliance has been placed on the *single* chemical analysis of a small quantity of food material withdrawn after an arbitrary one hour's sojourn in the stomach under examination. In our own experience, quite apart from the authoritative protests that have repeatedly come from both this and the other side of the Atlantic, there is no doubt that the old single test method led not a few of the profession astray in the more atypical gastric cases.

The technique of passing a very small gastric tube—or rather allowing it to be swallowed by the patient—is not difficult. Nor is it difficult or distressing for the patient to keep this tube in position for a considerable time. Withdrawal at short intervals of a fraction of the gastric contents and a series of analyses correlated on a time basis, obviously gives information of immensely greater value than can be obtained by the older single method.

Admitting that any test-meal examination is not always enjoyed by the patient dealt with in private practice, and that the procedure is therefore not employed as frequently as its text-book prominence would suggest, yet there seems little doubt that the practitioner has, in view of the inconclusive results previously obtained some excuse for his reluctance to employ it. Still if Dr. Ryle's most recent report be consulted, it is at once seen that by this more reasoned method the doctor is provided with a large amount of real clinical guidance. It is true that considerably more analyses must be made, but a result which really means something in the more obscure cases makes the trouble well repaid. For details the reader must be referred to the original clearly written paper, but we may in this note summarise the outstanding diagnostic points.

The method allows of an estimation of the rate of emptying at least as accurate as that by radiography and the barium meal. It allows of accurate qualitative and quantitive analyses of the resting gastric juice, as well as determination of its cytological and bacteriological characters. It makes possible the investigation of certain symptoms, such as "heartburn" and of pain having a special time relationship. The local action of drugs may be readily studied, and by means of parallel *x*-ray examination correlation of chemical and mechanical functions is easily possible. Macroscopic blood is also far easier of detection. But perhaps enough has been said to illustrate the great potentialities of this method, and we consider that the author reopened interest in an investigational channel which may have far-reaching effects upon both the clinical and research aspects of gastro-intestinal work.

